# The Tengmalm’s owl *Aegolius funereus* (Aves, Strigidae) as the definitive host of *Sarcocystis funereus* sp. nov. (Apicomplexa)

**DOI:** 10.3389/fvets.2024.1356549

**Published:** 2024-02-07

**Authors:** Ondřej Máca, Marek Kouba, Iva Langrová, Lucie Panská, Erkki Korpimäki, David González-Solís

**Affiliations:** ^1^Department of Pathology and Parasitology, State Veterinary Institute Prague, Prague, Czechia; ^2^Department of Zoology and Fisheries, Faculty of Agrobiology, Food and Natural Resources, Czech University of Life Sciences Prague, Prague, Czechia; ^3^Department of Ethology and Companion Animal Science, Faculty of Agrobiology, Food and Natural Resources, Czech University of Life Sciences Prague, Prague, Czechia; ^4^Department of Game Management and Wildlife Biology, Faculty of Forestry and Wood Sciences, Czech University of Life Sciences Prague, Prague, Czechia; ^5^Department of Ecology, Faculty of Environmental Sciences, Czech University of Life Sciences Prague, Prague, Czechia; ^6^Section of Ecology, Department of Biology, University of Turku, Turku, Finland; ^7^Department of Systematics and Aquatic Ecology, El Colegio de la Frontera Sur, Chetumal, Quintana Roo, Mexico

**Keywords:** rodents, birds of prey, Europe, sarcocysts, morphology, molecular characterization, oocysts and sporocysts, phylogeny

## Abstract

**Background:**

Owls have been reported as definitive hosts, whereas wild small mammals (naturally and experimentally) as intermediate hosts of several species of *Sarcocystis*. Recently, dead fledglings were found infected by an unnamed species of *Sarcocystis* since its intermediate host was unknown. After collecting additional samples of owls and wild small mammals, the present study focused on elucidating the identity, potential intermediate host, and complete life cycle of the found *Sarcocystis* through experimentally infected rodents. The developmental stages’ morphological and molecular characterizations (*28S* rRNA gene, ITS1 region) are presented herein.

**Methods:**

In total, 21 Tengmalm’s owl carcasses (15 nestlings, 5 fledglings, and 1 adult male) were collected in Kauhava (west-central Finland) and parasitologically examined by wet mounts. Intestinal mucosa scrapings were used to isolate oocysts/sporocysts and employed for experimental infections in dexamethasone-immunosuppressed BALB/cOlaHsd mice. Additionally, sarcocysts were searched in the skeletal muscle of 95 samples from seven wild small mammal species. All these developmental stages were molecularly characterized by the *28S* rRNA gene and ITS1 region. Experimental infections were carried out by using immunosuppressed female 8-week-old BALB/cOlaHsd mice, divided into three groups: (1) water with 15 μg/mL of dexamethasone, (2) water with 30 μg/mL of dexamethasone, (3) no dexamethasone treatment. Each group consisted of four individuals. In each group, two mice were infected with 1,000 sporocysts each, and the remaining two with 10,000 sporocysts each. All mice were euthanized on specific days post-infection.

**Results:**

The intestinal mucosa of 11 nestlings and 5 fledglings of the Tengmalm’s owl were positive for *Sarcocystis funereus* sp. nov. The adult male owl and all owls’ breast and heart muscles were negative for *Sarcocystis*. Two dexamethasone-immunosuppressed BALB/cOlaHsd mice (group 2) were positive to *S. funereus* sp. nov. in diaphragm and leg muscles after 22- and 24-day post-infection. Some sarcocysts were found in the wild small mammals. Molecular identification at *28S* rRNA revealed sequences from naturally infected Tengmalm’s owls, as well as sarcocysts of dexamethasone-immunosuppressed BALB/cOlaHsd mice were 99.87–100% similar to *Sarcocystis* sp. isolate Af1 previously found in the Tengmalm’s owl. At the ITS1 region, the *S. funereus* sp. nov. isolates Af2 haplotype B and Af3 haplotype A were 98.77–100% identical to *Sarcocystis* sp. isolate Af1. The sequences from sarcocysts of naturally infected wild small mammals were 75.23–90.30% similar at ITS1 region to those of *S. funereus* sp. nov.

**Conclusion:**

The morphological and molecular characterizations and phylogenetic placement of *S. funereus* sp. nov. are presented here for the first time and support the erection of the new species.

## Introduction

Species of *Sarcocystis* (Apicomplexa) have an indirect, two-host life cycle in which mammals, birds, reptiles, and humans are involved. Due to the wide variety of hosts acting as intermediate or definitive hosts, it is not easy to know the natural life cycle of many species. Birds represent a group of vertebrates commonly utilized as either intermediate and definitive hosts by various members of the genus *Sarcocystis*. Particularly, several species of owls have been reported as definitive hosts, while wild small mammals (rodents and shrews) serve as (natural and experimental) intermediate hosts of *Sarcocystis*. Such a relationship was, for instance, described in the case of the barn owl *Tyto alba* and the house mouse *Mus musculus* (1, 2) and the masked owl *Tyto novaehollandiae* (3) with *Sarcocystis dispersa*, the northern saw-whet owl *Aegolius acadicus* and the eastern deer mouse *Peromyscus maniculatus* with *Sarcocystis espinosai* (4), the tawny owl *Strix aluco* and *M. musculus* with *Sarcocystis scotti* [this species is considered invalid by Dubey et al. (5)] (3, 6, 7), *St*. *aluco* and the wood mouse *Apodemus sylvaticus* with *Sarcocystis sebeki* (8, 9), as well as the snowy owl *Bubo scandiacus* (reported as *Nyctea scandica*) and Richardson’s collared lemming *Dicrostonyx richardsoni* with *Sarcocystis rauschorum* (10, 11). Wiesner (12) further described an unnamed *Sarcocystis* species in the Tengmalm’s owl *Aegolius funereus* and experimentally found the bank vole *Clethrionomys* (=*Myodes*) *glareolus* to be its intermediate host. Thus, the involvement of wild small mammals in the life cycles of *Sarcocystis* parasitizing owls appears to be mandatory and obligate.

A recent analysis of a 45-year breeding data set on Tengmalm’s owl population in the Kauhava study area (Finland) revealed a decreasing trend in fledgling production corresponding with the long-term decline of the whole population (13). Additionally, we have documented long-term decline in the body condition of both male and females parent owls (14). Though the primary reasons for the observed trends regarding the local population are inappropriate forest management, we know practically nothing about the possible detrimental effects of different internal or external parasites on the individual Tengmalm’s owl’s long-term survival. The recent findings, indicating that *Sarcocystis* sp. isolate Af1 [see (15)] infected 100% of dead fledglings, have raised doubts about the presumed harmlessness of the mentioned parasite. Thus, the main aim of the present study was to determine the identity, potential intermediate host/s, and complete life cycle of the *Sarcocystis* sp. isolate Af1, through experimentally infecting rodents with newly collected oocysts and sporocysts isolated from the Tengmalm’s owls inhabiting the same study area. The morphological and molecular characterizations (*28S* rRNA gene, ITS1 region) of the developmental stages are presented herein.

## Methods

The Tengmalm’s owl carcasses were collected in the Kauhava study area (west-central Finland) throughout the breeding season 2021 during regular nest box visits starting in early April and later during radio-tracking of fledged young [see details in Kouba et al. (16)]. A total of 21 specimens (15 nestlings, 5 fledglings, and 1 adult male) were sent frozen to the State Veterinary Institute (SVI) Prague, Czech Republic, where parasitological examinations of intestine and muscles (breast, legs, and heart) were carried out by wet mounts. Intestinal mucosa scrapings were used to isolate oocysts/sporocysts under light microscopy with an optical microscope (Leica DM2500 LED), a digital camera (Leica DMC5400), and Leica Application Suite X microscope software (both Leica Microsystems, Wetzlar, Germany). Prior to experimental infections, 25 whole dead small mammals and 70 hind leg samples of seven wild species [i.e., the short-tailed field vole *Microtus agrestis* (2 whole bodies, 2 legs), the sibling vole *Microtus rossiaemeridionalis* (5 bodies), the European water vole *Arvicola amphibius* (1 body), the Eurasian harvest mouse *Micromys minutus* (5 bodies, 23 legs), the bank vole (4 bodies, 30 legs), the common shrew *Sorex araneus* (5 bodies, 15 legs), and the Eurasian pygmy shrew *Sorex minutus* (3 bodies)] were collected from owl nests and examined by wet smear of skeletal muscle. The prey items/bodies were exchanged for frozen newly hatched chickens not to deprive the owls of food.

Oocysts/sporocysts isolated from 2 birds were used to experimentally infect dexamethasone-immunosuppressed BALB/cOlaHsd mice (see below). At the same time, other parasite developmental stages were stored in Eppendorf tubes for DNA extraction under −20°C until further use. All measurements are given in micrometers unless otherwise specified. The molecular analysis of the oocysts/sporocysts isolates (*n* = 16) from the intestinal mucosa of owls and sarcocyst isolates from wild small mammals (*n* = 9) was carried out following that of Máca et al. (15), with minor changes. Genomic DNA of oocysts/sporocysts was extracted by the QIAamp® Fast DNA Stool Mini Kit (Qiagen, Hilden, Germany), while that of sarcocysts by using NucleoSpin tissue XS kit (Macherey-Nagel, Düren, Germany).

All isolates were characterized by the *28S* rRNA gene and ITS1 region by using the following primers: KL_P1F/KL_P2R, KL_P2F/P1R, and ITSR/ITSF, respectively (17). PCR procedures were performed in reaction mixtures consisting of 12.50 μL of GoTaq^®^ G2 Hot Start Green Master Mix (Promega, Madison, WI, United States), 0.4 μM of each primer, and 5 μL DNA template. RNase/Dnase-free water was used to top up the reaction mixture to a final volume of 25 μL. PCR amplification of negative controls was also conducted simultaneously. PCR conditions were as follows: 95°C for 3 min, 5 cycles of 94°C for 45 s, 64°C for 60 s, 72°C for 90 s; followed by 30 cycles of 95°C for 30 s, 58°C for 30 s, 72°C for 1 min, with a final elongation step of 72°C for 10 min. Amplified products were checked on 1% agarose gel electrophoresis and visualized on a UV transilluminator. Positive PCR products were purified with the ExoSAP-IT™ Express PCR Product Cleanup Reagent Kit (Thermo Fisher Scientific) and sent for sequencing on both strands (using the same forward and reverse primers as for the PCR) to the commercial company Eurofins Genomics (Ebersberg, Germany).

The reference nucleotide sequences used were selected based on similarities using the Basic Local Alignment Search Tool (BLAST) for sequence analysis^1^. The most similar sequences were downloaded and compared with the newly obtained sequences, aligned using the MAFFT software version 7 online server^2^ (18) for phylogenetic analysis using the MEGA 11 software version 11.0.13 (19). The phylogenetic trees were inferred by using the Maximum Likelihood (ML) with evolutionary distances calculated by the best-fitting model based on the lowest Bayesian Information Criterion (BIC) scores and resulted as Hasegawa-Kishino-Yano model (20) for the *28S* rRNA gene (analysis involved 38 nucleotide sequences with a total of 1,409 positions). The Hasegawa-Kishino-Yano model (20) was also the best model based on BIC scores for the ITS1 region (involving 25 nucleotide sequences with a total of 1,610 positions in the final dataset), both modeled with a Gamma distribution and invariant sites, with 1,000 bootstrap replications.

Immunosuppression of 8-week-old BALB/cOlaHsd female mice (ENVIGO) was required to establish a successful infection with *Sarcocystis* sporocysts. The mice were divided into three groups, each consisting of four individuals. Two of these groups received water-soluble dexamethasone sodium phosphate (Sigma-Aldrich) dissolved in their drinking water. The first group (*n* = 4) received water with a concentration of 15 μg/mL of dexamethasone continuously starting 2 weeks before the infection and continuing until the end of the experiment. The second group (*n* = 4) received water with a concentration of 30 μg/mL of dexamethasone 1 day before the infection until the end of the experiment. The third group (*n* = 4) did not receive any dexamethasone treatment. All mice were orally inoculated with oocysts/sporocysts delivered with food. In each group, two mice were infected with 1,000 sporocysts each, and the other two were infected with 10,000 sporocysts each. The data for parasitological examination was collected as follows: all mice were euthanized by intraperitoneal injection of ketamine (Narkamon 5%, Bioveta; 1.2 mL/kg) in combination with xylazine (Rometar 2%, Bioveta; 0.6 mL/kg) on specific days (22, 24, 52, 58, 69, and 77 days) after the infection with oocysts/sporocysts (Table 1). Subsequently, muscles containing sarcocysts were fixed in 10% formalin, embedded in paraffin, and sectioned. The histological sections were stained with hematoxylin and eosin and examined under the microscope (Leica DM2500 LED).

**Table 1:** Experimental design for establishing the *Sarcocystis* infection in 12 dexamethasone-immunosuppressed BALB/cOlaHsd mice (2 mice per treatment).

Treatment	Infection dose	Day of sacrifice
15 μg/mL	1,000 sporocysts	22, 58
15 μg/mL	10,000 sporocysts	58, 69
30 μg/mL	1,000 sporocysts	22, 22
30 μg/mL	10,000 sporocysts	22, 24
Without treatment	1,000 sporocysts	69, 77
Without treatment	10,000 sporocysts	52, 58

Owls were tagged and radio-tracked, and the carcasses were transported to Czechia under the approval of the Centre for Economic Development, Transport, and the Environment (Varsinais-Suomen Elinkeino-, Liikenne-ja Ympäristökeskus: permit numbers VARELY/1389/2018 and VARELY/5933/2019, respectively). All experiments and the maintenance of experimental animals were consistent with current animal welfare laws of the Czech Republic and were approved by the Animal Welfare Committee of the Czech University of Life Sciences Prague (permit number: MSMT-15824/2023-4).

## Results

The intestinal mucosa of 11 out of 15 (73%) Tengmalm’s owl nestlings and 5 out of 5 (100%) fledglings were positive for *Sarcocystis*. The only adult male owl available was negative to the presence of the parasite. The breast and heart muscles of all 21 examined owls were negative for *Sarcocystis*. No macroscopic lesions were observed in the organs of infected birds. No other protozoan gastrointestinal parasites were found. Sarcocysts from wild small mammals were found in 9 (1 *M. rossiaemeridionalis*, 7 *S. araneus*, 1 *S. minutus*) out of 95 samples (9.5% prevalence). All samples from *A. amphibius*, *C. glareolus*, *M. agrestis*, and *Mi*. *minutus* were negative for sarcocysts.

Developmental stages were described as follows:

Family Sarcocystidae Poche, 1913.

**Figure 1:**
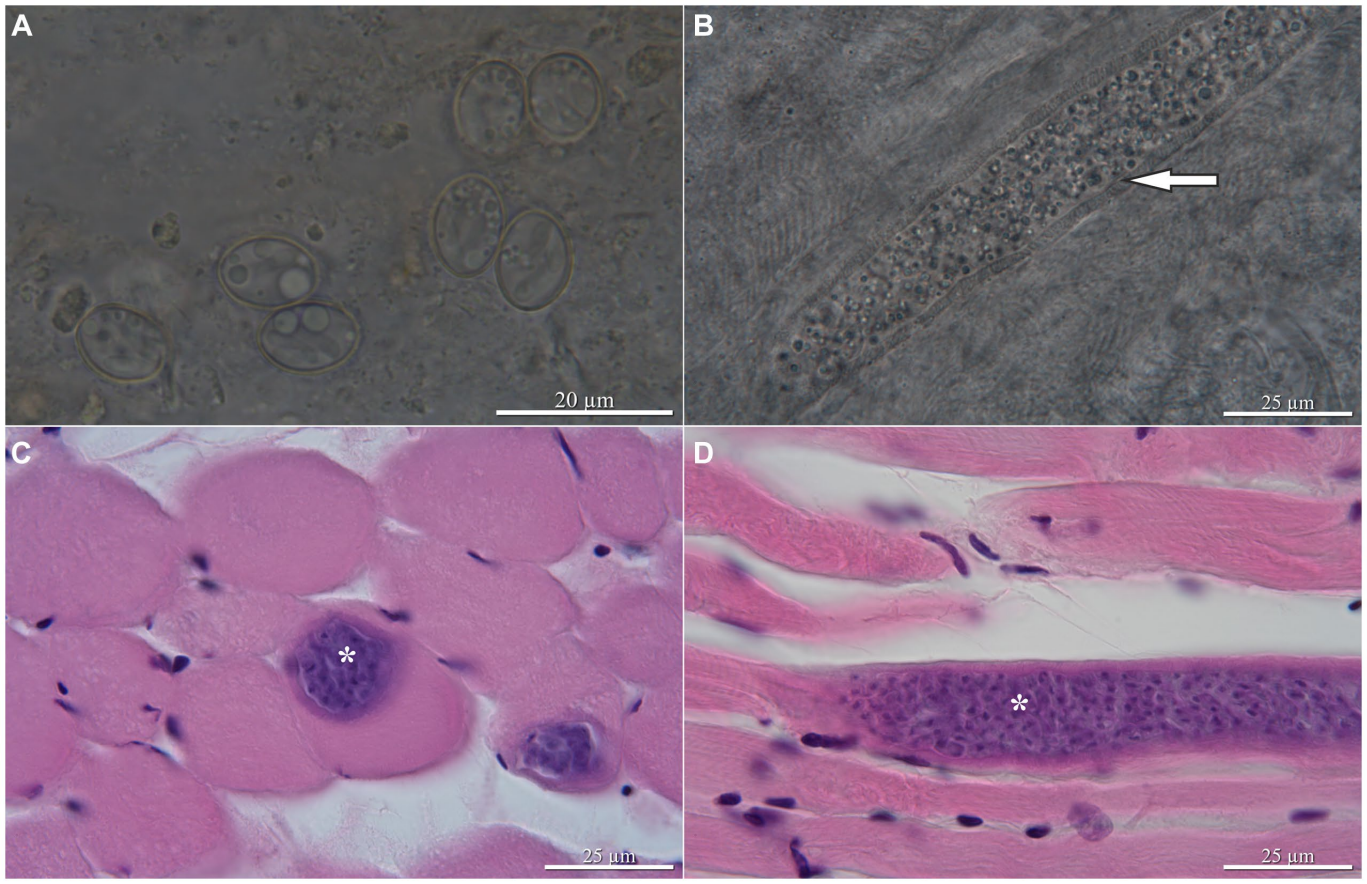
*Sarcocystis funereus* sp. nov., light micrographs. Oocysts/sporocysts from the intestinal mucosa of the Tengmalm’s owl *Aegolius funereus* from Finland; arrow indicates cyst wall with finger-like protrusions **(A)**, sarcocyst in wet mount **(B)** and hematoxylin and eosin staining **(C,D)** from the skeletal muscles of experimentally infected dexamethasone-immunosuppressed BALB/cOlaHsd mice 24 days postinfection, longitudinal and transversal sections, respectively. Asterisks indicate sarcocysts.

*Sarcocystis funereus* sp. nov. (Figure 1).

Description: Thin-walled sporulated oocysts, 16.7–17.0 × 11.5–12.2 (*n* = 5), and sporocysts (Figure 1A) were 11.3–12.9 × 8.1–9.3 (*n* = 50). Sarcocysts were microscopic, the largest 219.5 long and 30.7 wide (found at 24 days post-infection), elongate, ribbon-shaped, with both ends rounded (Figures 1B–D). Wall was 3.1 thick, characterized by dense finger-shaped villar protrusions, 2.6 long (Figure 1B).

### Taxonomic summary

Definitive host: Tengmalm’s owl *Aegolius funereus* Linnaeus, 1758 (Strigiformes: Strigidae).

Natural Intermediate host: Unknown.

Experimental intermediate host: Dexamethasone-immunosuppressed BALB/cOlaHsd mouse.

Distribution: Kauhava region, west-central Finland (~63° N, 23° E).

Site of infection: Small intestine (definitive host), skeletal muscle (experimental intermediate host).

Deposited material: Symbiotype (oocysts/sporocysts in 2.5% potassium dichromate) and genomic DNA in an Eppendorf tube were stored at SVI Prague. GenBank sequences OR725602 and OR726006 (*28S* rRNA gene), OR726007 and OR726008 (ITS1 region). Positive mice were frozen at −20°C, and histology slides were stored in lab SVI Prague.

Sequences obtained from the experimental study: OR725602 (*28S* rRNA gene), and OR726007 (ITS1 region).

ZooBank registration: To comply with the regulations in article 8.5 of the amended 2012 version of the International Code of Zoological Nomenclature (21), details of the new species have been submitted to ZooBank. The Life Science Identifier (LSID) for *Sarcocystis funereus* sp. nov. is urn:lsid:zoobank.org:pub:536F1351-1157-4C09-999A-D41078EE3CBC.

Etymology: The specific epithet is derived from the species name of its definitive host, i.e., *funereus*.

Molecular identification at *28S* rRNA revealed that 12 sequences [isolate Af3 haplotype A (OR726006) and isolate Af2 haplotype B (OR725602), both 1,509 bp] obtained from the 16 oocyst/sporocyst isolates (4 failed sequencing) from the naturally infected Tengmalm’s owls, as well as the 6 sequences (haplotype B) of the 6 sarcocyst isolates in the skeletal muscles of dexamethasone-immunosuppressed BALB/cOlaHsd mouse were 99.87–100% similar to *Sarcocystis* sp. isolate Af1 (MW349707), 97.59% similar to *Sarcocystis strixi* (MF162316) and 97.46–97.53% to *Sarcocystis lari* (MF946611), in the white-tailed sea eagle *Haliaeetus albicilla* from Norway; and 97.42–97.49% similar to *Sarcocystis lutrae* (KM657771) in the Eurasian otter *Lutra lutra* from Norway. Haplotypes A and B were 99.87% similar each other and showed single cases of double peaks at nucleotide positions 666 and 667 (TT/CC), especially in those samples from owls and experimental mice that resulted in TT (haplotype A) or CC (haplotype B) peaks or double peaks at these positions. Isolates used for experimental infections and those of all sarcocysts resulted in CC dominant peak at this position and represent *S. funereus* sp. nov. isolate Af2 haplotype B (OR725602). *Sarcocystis* cf. *strixi* isolate LTAfl120 (OQ557459) in the tawny mouse *Apodemus flavicollis* from Lithuania and *Sarcocystis* sp. isolate No. 5 (AF513497) in *S. araneus* from Czech Republic were 95.37 (47% query cover; 733 bp) and 96.22% (35% query cover; 554 bp), respectively, similar to haplotypes A and B of *S. funereus* sp. nov., although both have short sequences and were not used in the phylogenetic analysis.

All isolates were successfully sequenced at the ITS1 region. Like at the *28S* rRNA gene, the isolates *S. funereus* sp. nov. isolate Af2 (OR726007, 1,300 bp) and *S. funereus* sp. nov. isolate Af3 (OR726008, 1,297 bp) were used for infection and all sequences obtained from sarcocysts (OR726007) showed 10 SNPs and 3 nucleotide insertions of GTG in position 1,011–1,013. Those insertions were not found in other ITS1 region sequences that Máca et al. (15) obtained. Similarly, there was only one SNP (T/C) at nucleotide position 466 in newly obtained sequences. Moreover, *S. funereus* sp. nov. isolate Af2 (OR726007) and *S. funereus* sp. nov. isolate Af3 (OR726008) were 98.77–100% identical to *Sarcocystis* sp. isolate Af1 (MW373964), and 88.43–89.57% (26–45% query cover) to *S. lutrae* (MG372108) in the European badger *Meles meles* from the Czech Republic and *Sarcocystis halieti* (MF946596) in the white-tailed sea eagle from Norway.

**Figure 2:**
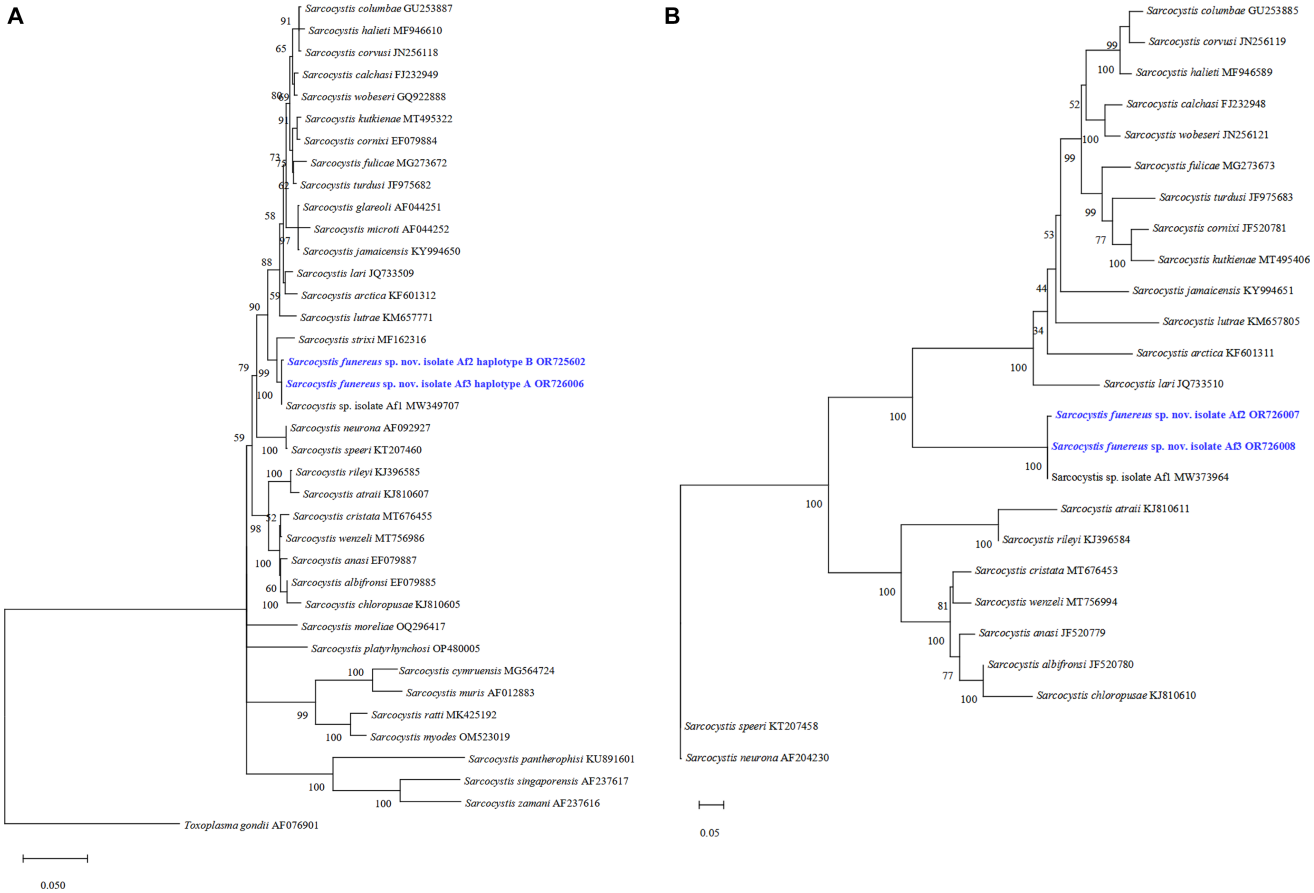
Phylogenetic trees of the related species of *Sarcocystis* from various hosts based on sequences of the *28S* rRNA gene **(A)** and the ITS1 region **(B)**. The numbers on phylogenetic trees represent bootstrap values based on 1,000 replications. GenBank accession numbers follow *Sarcocystis* taxa.

The phylogenetic tree showed that isolates Af3 haplotype A and Af2 haplotype B of *S. funereus* sp. nov. at the *28S* rRNA gene grouped with *Sarcocystis* sp. Af1, which was previously reported in *A. funereus*. Moreover, *S*. *strixi* appears as a sister species in a close clade (Figure 2A). At the ITS1 region, sequences of haplotypes A and B are also grouped in a single clade with *Sarcocystis* sp. Af1 (Figure 2B).

Out of the 8 dexamethasone-immunosuppressed BALB/cOlaHsd mice used for the experimental infections, only 2 of the second group, treated with 30 μg/mL dexamethasone and dosed with 10,000 sporocysts were positive to *S. funereus* sp. nov. after 22- and 24-day post-infection. Both mice showed sarcocysts in the skeletal muscles (diaphragm, leg), but no in the brain nor heart. Infected mice were asymptomatic during the whole infection process, from inoculation to euthanasia.

The 6 sequences (5 from *S. araneus*, 1 from *S. minutus*) successfully obtained from the sarcocysts of naturally infected small mammals were molecularly identified by using the ITS1 region marker (unpublished data). Those sequences from *S. araneus* and *S. minutus* were 75.23–90.30% (13–26% query cover) and 90.30% (19% query cover), respectively, similar to *Sarcocystis* sp. isolate Af1 (MW373964) and *S. funereus* sp. nov. isolate Af3 (OR726008). Negative PCR resulted in the samples of *M. rossiaemeridionalis* and 2 of *S. araneus*.

## Discussion

While recently reporting the finding of oocysts/sporocysts in the intestine of the Tengmalm’s owl, Máca et al. (15) refrained from specifically naming the *Sarcocystis* sp. isolate Af1, since its intermediate host and sarcocysts were unknown. This unnamed *Sarcocystis* was fully characterized by using four loci (*18S* rRNA, *28S* rRNA, ITS1, and *CO1*), so we decided to only use two of them (*28S* rRNA and ITS1) because both, but especially the ITS1 region, clearly delimite species using avian hosts than the other two loci (22). Máca et al. (15) mentioned that the ITS1 region is more sensitive to the genetic differences among *Sarcocystis* species from birds and carnivores as intermediate hosts, while CO1 are considered of limited taxonomic help.

After the experimental infections of mice, the presence of sarcocysts in the skeletal muscles elucidates the determination of such species as new to science and the possible route of the life cycle. The current finding supports the rodent-owl life cycle reported in our previous study (15). The phylogenetic position of *S. funereus* sp. nov. inferred separately at the *28S* rRNA gene and ITS1 region was the same than that obtained by Máca et al. (15); thus, *Sarcocystis* sp. isolate Af1 should be considered to belong to *S. funereus* sp. nov.

There is only one previous record of an unnamed *Sarcocystis* in the Tengmalm’s owl, whose sporocysts and oocysts were not morphologically nor molecularly described [see (12)]; additionally, this unnamed species was experimentally transmitted to the bank vole, while the new species to immunosuppressed mice. Considering that *Sarcocystis* are more specific to their rodent intermediate hosts [see (5)] and that those bank voles examined during this study were free of sarcocysts, they most probably represent two different species. Our opinion supports the finding that *S*. *sebeki* of the house mouse was not transmissible to the wood mouse, bank vole or meadow vole (*Microtus arvalis*) (9).

The Tengmalm’s owl acts as the definitive host for *Sarcocystis* sp. Wiesner, 1980 [as named by Levine and Ivens (23)] and *S. funereus* sp. nov., with bank voles and mice, respectively, are its intermediate hosts. However, more information on the former unnamed species is required. The finding of *S*. *halieti* and *S*. *lari* in the white-tailed sea eagle showed that more than one *Sarcocystis* species might infect a particular bird of prey species [see (22)]. As stated by Máca et al. (15), more Tengmalm’s owls and other birds of prey species should be examined to determine the presence of other parasite species or morphospecies of *Sarcocystis*.

Corticosteroids, such as dexamethasone, have proven to be useful in developing animal models for studying coccidian parasites (24). Dexamethasone induces the depletion of CD4+ T lymphocytes (25) and suppresses T- and natural killer (NK) cell-mediated immunity (26). Similarly, Interferon-gamma (IFN-gamma) gene knockout (KO) mice, often employed to establish *Sarcocystis neurona* infection in laboratory mice, lack a CD4+ Th1 response. The immunity of gamma-IFN KO mice is either deleted or reduced, allowing the establishment of infection and the development of clinical disease in mice (27). Therefore, we selected dexamethasone immunosuppression to set up *Sarcocystis* infection. The optimal concentration of dexamethasone in the drinking water was determined to be 30 μg/mL, as a lower concentration likely did not sufficiently suppress the immune response for successful infection establishment.

Máca et al. (15) found *S*. *strixi* to cluster with *Sarcocystis* sp. isolate Af1 (now recognized as *S. funereus* sp. nov.) and regarded them as distinct species. Interestingly, both have a densely covered wall of the sarcocyst, although *S*. *strixi* has knob-like blebs and a thinner wall (< 2 μm). In contrast, *S. funereus* sp. nov. has longer finger-shaped blebs and a thicker wall (> 2 μm). Additionally, the ends of the sarcocyst (pointed vs. rounded) differ from each other.

Six sequences from wild small mammals were positive for *Sarcocystis*, but none of them could be considered as *S. funereus* sp. nov. They probably belong to one or several different species, although more analyses are needed to fully understand whether they represent various species and find their natural definitive hosts. The natural intermediate host of *S. funereus* sp. nov. is still unknown, but it is very likely that the Eurasian harvest mouse, one of the most common small mammals used as prey by the Tengmalm’s owls (28, 29), plays that important role. Interestingly, after examining several samples of this host (*n* = 28), no sarcocysts were found. It does not mean that they cannot be parasitize by *Sarcocystis*, but that the density of intermediate hosts, visiting of sites with contaminated feces, and the climatic seasons are important factors to determine the infection of the definitive hosts, as stated by Hoogenboom and Dijkstra (30). Therefore, further investigations of potential intermediate hosts (wild small mammals) should be done to warrant the identification of the real host. As in other species of *Sarcocystis* using owls as definitive hosts, mice are essential in the life cycle, as *S*. *espinosai* in the northern saw-whet owl and eastern deer mouse [see (4)].

The present findings show the role of the Tengmalm’s owl in the life cycle of a *Sarcocystis* species and increase our knowledge of rodent predators as part of the life cycle of parasites. Many more studies are needed to understand the individual relationships between parasites and their intermediate and final hosts. As in other parasite/birds of prey relationships worldwide, we know nothing about the impact of *S. funereus* sp. nov. on the body condition of fledglings, dispersal, and probability of recruitment to the breeding population and particularly long-term survival of the Tengmalm’s owl. It is important to understand if it is possible to have intestines full of parasites without adversely affect the health of individuals, as in the present study. Thus, more focus and studies are needed to determine how parasites and their hosts interact and influence each other’s lives, either negatively or positively.

## Conclusion

This work elucidates the specific identity of the *Sarcocystis* infecting the Tengmalm’s owl and its experimental intermediate host, although more prey and potential intermediate hosts should be examined to determine their role in the life cycle of the new species. The morphological and molecular characterizations and phylogenetic placement of *S. funereus* sp. nov. are presented here for the first time and support the erection of the new species.

## Data availability statement

The datasets presented in this study can be found in online repositories. The names of the repository/repositories and accession number(s) can be found in the article/supplementary material.

## Ethics statement

The animal study was approved by the Varsinais-Suomen Elinkeino-, Liikenne-ja Ympäristökeskus (permit numbers VARELY/1389/2018 and VARELY/5933/2019), respectively, and welfare laws of the Czech Republic and were approved by the Animal Welfare Committee of the Czech University of Life Sciences Prague (permit number: MSMT-15824/2023-4). The study was conducted in accordance with the local legislation and institutional requirements.

## Author contributions

OM: Conceptualization, Data curation, Formal analysis, Funding acquisition, Investigation, Methodology, Project administration, Resources, Software, Supervision, Validation, Visualization, Writing – original draft, Writing – review & editing. MK: Conceptualization, Funding acquisition, Investigation, Methodology, Writing – review & editing. IL: Formal analysis, Investigation, Methodology, Supervision, Writing – review & editing. LP: Investigation, Methodology, Writing – review & editing. EK: Investigation, Methodology, Writing – review & editing. DG-S: Conceptualization, Formal analysis, Supervision, Writing – original draft, Writing – review & editing.

## Footnotes

^1^ https://blast.ncbi.nlm.nih.gov/Blast.cgi

^2^ http://mafft.cbrc.jp/alignment/software/
